# Extent of Anxiety Among Married Women in Bangladesh and its Potential Predictors: A Nation-Wide Cross-Sectional Study

**DOI:** 10.1155/da/7415491

**Published:** 2025-09-22

**Authors:** Mortuja Mahamud Tohan, Sayeeda Zaman, Paumpy Paul, Nasrin Sultana, Abu Sadat Nurullah, Md. Ashfikur Rahman

**Affiliations:** ^1^School of General Education, BRAC University, Dhaka, Bangladesh; ^2^Development Studies Discipline, Social Science School, Khulna University, Khulna, Bangladesh; ^3^Department of Applied Social Sciences, Faculty of Health and Social Sciences, The Hong Kong Polytechnic University, HKSAR, China

**Keywords:** anxiety, Bangladesh, married women, socioeconomic

## Abstract

Anxiety is a significant mental health challenge for women of reproductive age worldwide, often contributing to broader psychological issues. However, research on anxiety prevalence among this demographic, particularly in Bangladesh, remains limited. This study addresses this gap by identifying potential predictors of anxiety among married women in Bangladesh. Using data from the nationally representative Bangladesh Demographic and Health Survey (BDHS) 2022, the generalized anxiety disorder (GAD-7) scale was employed to assess anxiety levels. Descriptive and inferential statistical analyses, including one-way ANOVA and stepwise multiple regression, were conducted to identify key predictors. The findings reveal that 25.8% of married women in Bangladesh experience mild to severe anxiety, with 4.1% reporting moderate to severe anxiety. A six-factor model derived from stepwise multiple regression explained 17.3% of the variance in anxiety levels. The most significant predictor was a history of terminated pregnancy, accounting for 6.8% of the variance (*R*^2^ change = 0.068; *p* ≤ 0.001). Other notable predictors included pressure from spouses or family members (*R*^2^ change = 0.038; *p* ≤ 0.001), educational status (*R*^2^ change = 0.028; *p* < 0.001), religion (*R*^2^ change = 0.019.; *p*=0.018), continuation of education after marriage (*R*^2^ change = 0.012; *p*=0.030), and husband's educational attainment (*R*^2^ change = 0.007; *p*=0.033). Additional factors such as employment after marriage, age at first sexual intercourse, and wealth status also played significant roles. The study highlights the substantial prevalence of anxiety among married women in Bangladesh, emphasizing the influence of socioeconomic along with other potential factors. Further research is needed to develop targeted interventions addressing socioeconomic and behavioral determinants, ensuring the mental well-being of married women.

## 1. Introduction

Anxiety is associated with a distinct mode of cognitive functioning that influences processing priorities. Specifically, it is characterized by hypervigilance, where individuals scan their environment for threat-related stimuli, heightening the initial encoding of potential threats and shaping cognitive processing accordingly [1]. This psychological mechanism is particularly relevant when examining anxiety among women in developing countries like Bangladesh, where patriarchal social structures perpetuate gender inequalities that affect mental health [2]. The intersection of patriarchal norms, economic dependency, and rigid gender roles exposes women to heightened levels of stress, anxiety, and depression, particularly married women who face an added burden of domestic and familial responsibilities.

Married women represent a substantial proportion of Bangladesh's population [3]. Despite their significant demographic presence, their mental health struggles remain underexplored and unsupported. Structural inequalities, exacerbated by gender stereotypes and financial dependance on men, intensify their psychological distress. Married women experience higher rates of anxiety and depression than their single counterparts due to socioeconomic pressures, unpaid labor, and societal expectations [4]. Furthermore, financial insecurity, limited employment opportunities, and the dual burden of work and caregiving significantly contribute to chronic stress and low self-esteem.

The World Health Organization (WHO) highlights those women in low- and middle-income countries (LMICs) are disproportionately affected by mental health issues due to gender-specific challenges, including economic dependency and restrictive social norms [5]. In Bangladesh, mental health issues—especially those stemming from socioeconomic stressors—are often stigmatized, leading to a lack of recognition and timely intervention [6]. Married women, regardless of their employment status, encounter significant challenges that contribute to anxiety. Those engaged in unpaid domestic work experience social isolation and financial insecurity, meanwhile those employed outside the home struggle with balancing professional responsibilities with traditional caregiving roles. Both groups contend with economic instability, which further amplifies their psychological distress.

Existing literature emphasizes the strong relationship between socioeconomic factors and mental health. Research has consistently shown that lower income, limited education, and unemployment increase the risk of anxiety and depression among married women [7]. Studies have revealed that married women report higher stress levels than single women, with 33% of married women experiencing significant stress compared to 22% of single women [8]. Moreover, factors such as marital satisfaction, gender roles, and social expectations significantly influence depression and anxiety in married adults [9]. Chowdhury [10] found that supportive marriages improve women's mental health, while conflicted marriages with poor communication aggravate anxiety and depression. Similarly, research on married women in Egypt demonstrated a high prevalence of depression linked to low income, limited education, and high household responsibilities [11].

In South Asia, patriarchal norms and economic dependency are major contributors to women's mental health struggles. Lamichhane et al. [12] identified that low social status, limited education, and economic dependence increase the risk of violence against young married women in rural Nepal, further worsening their mental health outcomes. Recent studies have reported a 4%–19.5% prevalence of anxiety symptoms among reproductive-aged women in Bangladesh, with significant predictors including older age, regional disparities, household decision-making autonomy, lower educational attainment, and extremes in body mass index (BMI) [13–16]. However, these studies often treat women as a homogeneous group, overlooking the distinct mental health challenges married women face due to compounded caregiving burdens, social expectations, and economic dependency. Additionally, localized research on early-married adolescents highlights links between IPV and anxiety but lacks national generalizability [17]. While these studies offer valuable insights, there is a lack of focused research on the unique mental health challenges faced by married women in Bangladesh. This gap highlights the need for a comprehensive investigation into the intersection of socioeconomic conditions and their mental well-being.

This study addresses this gap by offering the nationally representative analysis focusing exclusively on married women in Bangladesh, highlighting how their structural and gendered realities influence anxiety. This focus provides new insights for developing targeted, gender-responsive mental health strategies within the Bangladeshi context. The findings will guide the formulation of policies that promote financial independence, enhance decision-making abilities, and improve mental well-being. Ultimately, this study seeks to contribute to optimizing family dynamics, fostering societal well-being, and encouraging gender-responsive policies that support the mental health of married women in Bangladesh.

## 2. Materials and Methods

### 2.1. Data Source and Collection Technique

The data for this study were drawn from the 2022 Bangladesh Demographic and Health Survey (BDHS), a nationally representative, cross-sectional survey conducted by the National Institute of Population Research and Training (NIPORT), in collaboration with ICF and Mitra and Associates. The survey employed a stratified two-stage cluster sampling design based on the 2011 Bangladesh Population and Housing Census, with sampling stratified by urban/rural residence and administrative division. In the first stage, 672 enumeration areas (EAs) were randomly selected with probability proportional to size. In the second stage, 30 households were systematically selected from each EA, resulting in a sample of approximately 20,160 households [18].

Data collection took place between January and June 2022 and was carried out by trained female interviewers using standardized, pretested questionnaires in Bangla. Interviews were conducted face-to-face using tablet-based data entry systems. For the mental health module, including the generalized anxiety disorder (GAD-7) scale and the Water Glass Pictorial Scale, respondents were married women aged 15–49 years. Informed verbal consent was obtained from all participants prior to participation, in accordance with ethical standards approved by the Bangladesh Medical Research Council (BMRC) and the ICF Institutional Review Board (IRB). Sampling weights provided by the DHS program were applied in all analyses to ensure representativeness at the national and regional levels and to correct for differences in probability of selection and response rates. The demographic composition of the study sample was broadly consistent with national benchmarks reported in the 2022 BDHS Final Report.

### 2.2. Outcome Measure

This study employed the generalized anxiety disorder (GAD-7) scale, a widely recognized tool for measuring anxiety levels across diverse populations [19]. The GAD-7 scale was integrated into the mental health module of the 2022 BDHS to assess anxiety among married women in Bangladesh. The scale, which has previously been validated in the Bangladeshi context, evaluates symptoms experienced during the 2 weeks preceding the survey [20]. Scores are categorized as mild (0–5), moderate (6–14), and severe (15–21) anxiety [21]. Responses are measured using a Likert scale with the options “not at all,” “several days,” “more than half the days,” and “nearly every day,” which are assigned numerical values (0, 1, 2, and 3, respectively) to quantify symptom severity. The final score is derived by summing the scores for all items, and those items demonstrated good reliability (Cronbach's alpha = 0.833). The 2022 BDHS utilized the validated Water Glass Pictorial Scale to assess symptom intensity. This scale uses visual representations, where an empty glass signifies “never,” a one-fourth-full glass represents “rarely,” a half-full glass indicates “often,” and a three-fourths-full glass denotes “always.” This scale was used concurrently with the GAD-7 to facilitate more accurate and meaningful responses, particularly in rural or low-literacy populations where abstract Likert-type scales may be difficult to interpret. By providing a visual aid aligned with each GAD-7 response option, the Water Glass Pictorial Scale helped bridge cognitive gaps, minimize response bias, and improve data quality. One notable limitation of this study is the absence of clinical diagnostic testing for anxiety. As the analysis was based on secondary data from the 2022 BDHS, we were limited to using the GAD-7 scale as a screening tool rather than a diagnostic instrument. Consequently, the study cannot definitively classify respondents as having clinical anxiety; rather, it reflects self-reported symptoms indicative of varying anxiety severity levels.

### 2.3. Predictor Variables

To identify predictor variables associated with the outcome measures, a comprehensive literature review was conducted. Based on the available dataset and existing research, a range of biological, socioeconomic, and behavioral variables were selected for analysis (Table 1). Socioeconomic variables included age, region, educational attainment, wealth status, residence type (urban/rural), religion, and employment status. Biological and behavioral factors included age at first sexual intercourse, history of terminated pregnancies, and use of contraceptives, smartphone ownership, and internet usage. Additionally, social factors such as the decision-making autonomy regarding contraceptive use, pressure to conceive, and autonomy in visiting healthcare facilities were considered as potential predictors. These variables were selected based on their relevance to understanding the factors influencing anxiety levels among the target population.

### 2.4. Statistical Analysis

Both descriptive and inferential statistical analyses were employed in this study. Descriptive statistics were utilized to summarize the background characteristics of the respondents, providing an overview of their demographic and socioeconomic profiles. Inferential statistics were applied to identify the predictor variables associated with the outcome measures, specifically focusing on anxiety among married women in Bangladesh. One-way ANOVA was performed to examine mean differences in anxiety scores across categories of independent variables. Then stepwise multiple regression analysis was conducted to estimate the simultaneous examination of multiple predictor variables to understand their collective impact on the outcome variable. Variables were entered into the model based on a stepwise selection method, using an entry *p*-value threshold of <0.05 and a removal threshold of >0.10. This approach ensured that only variables with statistically significant contributions to the model were retained.

All data analyses were performed using STATA MP 16 and SPSS 26 software packages.

## 3. Result

### 3.1. Background Characteristics

Table 2 illustrates that, out of 28,537 participants, the majority belonged to the age group of 35–49 years (61.1%), with a significant proportion residing in rural areas (65.1%). Over half of the women (61.2%) had attained secondary or higher education, while 42.6% belonged to rich households. Additionally, the majority of their husbands (50.9%) had completed secondary or higher education. Notably, a significant proportion of women (80.7%) discontinued their studies after marriage, while 51.4% did not continue working after marriage. Approximately one third (30.1%) of the women were employed, and the majority of respondents (89.5%) identified as Muslim. Among the respondents, 69.2% owned a mobile phone, while 46.4% had a smartphone. In terms of internet usage, 70.5% had never used the internet.

The majority of women (65.4%) reported having their first sexual encounter before the age of 18, while 38.9% had their first birth before 18. Approximately one-quarter (22.6%) had a history of terminated pregnancy. Most respondents (84.7%) utilized some form of contraception to delay or avoid pregnancy. In terms of decision-making, the majority (89.8%) reported that they made choices about contraceptive use either alone or with their partner. A similar pattern was observed for healthcare visits (74.3%) and visiting relatives (73.7%), with respondents making these choices independently or with their partner. Furthermore, a small percentage (3.1%) indicated that they had felt pressured by their husband or family members to become pregnant.

Based on the GAD-7 criteria, approximately three out of four women (74.2%) experienced minimal anxiety, while 21.7% had mild anxiety. The prevalence of moderate anxiety was notably low at 3.2%, and only 0.9% of respondents reported severe anxiety.

### 3.2. Prevalence of Anxiety Among Married Women

Table 3 presents the distribution of anxiety symptoms among married women in Bangladesh based on responses to the GAD-7 scale. The findings show that the majority of respondents did not frequently experience anxiety-related symptoms in the 2 weeks prior to the survey. For most items, more than half of the women reported “never” experiencing the symptom—ranging from 53.8% for “worrying about things” to 83.9% for “feeling afraid as if something awful might happen.” The only item where “rarely” surpassed “never” was “feeling nervous, anxious, or on edge,” with 47.2% choosing “rarely” and 41.7% selecting “never.” Reports of more persistent symptoms were relatively low, with less than 3.3% of respondents selecting “often” and under 1.5% choosing “always” across all items. The highest combined percentage of “often” and “always” responses (11.1%) was observed for feeling nervous or on edge, while the lowest (2.6%) was noted for feeling afraid something terrible might happen. Overall, the data indicate that while occasional anxiety symptoms were somewhat common, sustained or severe anxiety was reported by only a small portion of the women surveyed.

### 3.3. Potential Factors Linked to anxiety

Table 4 presents the results of the one-way ANOVA, indicating that participants aged 35–49 years reported significantly higher anxiety symptoms (mean GAD score = 3.08; *p*  < 0.011) in comparison with other age categories. The higher level of anxiety scores was observed among participants with no education (mean GAD score = 3.72; *p* < 0.001) or those who identified as Muslim (mean GAD score = 3.10; *p* < 0.001). It was also observed that women from poor households (mean GAD score = 3.27; *p* < 0.001) were more likely to develop anxiety. Additionally, respondents who reported to have their first sexual encounter before the age of 18 (mean GAD score = 3.13; *p* < 0.001); or those who had their first birth before 18 (mean GAD score = 3.22; *p* < 0.001) exhibited significantly greater anxiety symptoms.

Respondents who were currently employed reported a significantly higher anxiety score (mean GAD = 3.17; *p* < 0.001) compared to those who were unemployed. Participants who did not own a smartphone had significantly higher scores for anxiety (mean GAD = 3.27; *p* < 0.001). Similarly, individuals who had never used the internet scored higher anxiety scale (mean GAD = 3.21; *p* < 0.001) compared to those who had. Women who had experienced a terminated pregnancy reported significantly higher anxiety (mean GAD = 3.48; *p* < 0.001).

Respondents whose husbands had no formal education (mean GAD = 3.59; *p* < 0.001) Furthermore, respondents who reported being pressured by their husbands or family members to become pregnant (mean GAD = 4.47; *p* < 0.001); discontinued their studies after marriage (mean GAD = 3.06; *p* < 0.001); made decisions regarding healthcare visits alone or with their partner (mean GAD = 3.13; *p* < 0.001); made decisions about visiting relatives' houses alone or with their spouse (mean GAD = 3.14; *p* < 0.001) reported higher anxiety.

While the one-way ANOVA results revealed statistically significant differences in GAD-7 scores across several demographic and socioeconomic groups, it is important to note that the actual magnitude of these differences was modest. For example, although respondents from poor households and those with no formal education reported higher anxiety scores, the average differences were often less than one point on the 21-point GAD-7 scale. These small mean differences may not be clinically significant at the individual level. However, from a population health perspective, even modest increases in anxiety scores across large subgroups can translate into meaningful public health concerns, particularly in resource-limited settings like Bangladesh.

### 3.4. Stepwise Multiple Regression

A stepwise multiple regression (Table 5) analysis has identified a six-factor model that reports 17.3% of the variance of anxiety among married women in Bangladesh. The most significant predictor of anxiety is having ever experienced a terminated pregnancy, which explains 6.8% of the variance (*R*^2^ change = 0.068; Sig. ≤ 0.001). Pressure from spouses or family members to become pregnant is the second most influential predictor, accounting for an additional 3.8% of the variance (*R*^2^ change = 0.038; Sig. ≤ 0.001). Additionally, respondents' educational status ranks third, accounting for 2.8% of the variance (*R*^2^ change = 0.028; Sig. ≤ 0.001). The fourth factor is religion, which explains 1.9% of the variance (*R*^2^ change = 0.019; Sig. = 0.018), while continuation of study after marriage represents the fifth predictor accounting for 1.2% of the variance (*R*^2^ change = 0.012; Sig. = 0.030). Last, husband's educational attainment explains an additional 0.7% of the variance (*R*^2^ change = 0.007; Sig. = 0.033). This model underscores key psychosocial and demographic factors associated with anxiety, with the termination of pregnancy being identified as the strongest predictor.

## 4. Discussion

This study aimed to shed light on the socioeconomic predictors of anxiety among married women in Bangladesh, contributing to the growing literature on mental health dynamics in LMICs. Based on the analysis of the 2022 BDHS, this research identified a range of demographic and socioeconomic factors associated with elevated anxiety symptoms, as measured by the GAD-7 scale. The decision to rely on one-way ANOVA, rather than logistic regression models, was deliberate: given the limited variance explained by potential predictors and the cross-sectional nature of the data, the analysis focused on identifying statistically significant differences in mean anxiety scores across various subgroups. Variables included in the analysis were selected based on prior empirical findings, theoretical relevance, and availability within the BDHS dataset.

The most significant predictor of anxiety identified in this study was having experienced a terminated pregnancy. This finding is consistent with prior research conducted in Bangladesh and Nepal, demonstrating a strong correlation between pregnancy loss and mental health challenges among reproductive-aged women [22, 23]. Research indicates that the psychological consequences of pregnancy loss can be influenced by various factors, including age, gestational stage at the time of loss, religious and cultural beliefs, social stigma, self-efficacy, family relationships, and social support [24–26]. For some women, the emotional toll of a terminated pregnancy may lead to prolonged anxiety, which could potentially affect subsequent pregnancies [27, 28]. These findings emphasize the need for comprehensive psychosocial support systems to meet the mental health needs of women who have experienced pregnancy loss.

In line with previous studies, age has been identified as a significant demographic factor influencing anxiety, with women in the 35–49 age group being more likely to experience anxiety compared to younger age groups [23, 29, 30]. Women in mid-age are often balancing multiple responsibilities, managing households' activities, caregiving for children and elderly family members, and maintaining employment may impose additional burdens [31–33]. Notably, this demographic is more likely to experience age-related fertility issues, perimenopausal symptoms, and concerns regarding future health, which can exacerbate feelings of anxiety [34–36]. Contrarily, several studies found that younger reproductive-age populations report higher levels of anxiety symptoms compared to older groups [37, 38]. This study reported that Muslim women experience higher levels of anxiety compared to followers of Hinduism or other religions, which aligns with findings from a similar study conducted in Bangladesh [39]. This may be attributed to a combination of social stigma and stricter gender expectations, which can contribute to the development of anxiety. Nevertheless, while Islam advocates for the respect of women's roles and rights, the oppressive situations faced by Muslim women often arise from misinterpretations of its principles [40, 41].

Consistent with prior research [23, 42, 43], socioeconomic status emerged as a critical determinant of anxiety, with women from low socioeconomic households exhibiting significantly higher levels of anxiety compared to those from more affluent backgrounds. This is because financial hardship, a hallmark of poverty, generates chronic stressors such as food insecurity, inadequate housing, and inability to afford healthcare—factors that directly intensify anxiety [44, 45]. In low-resource settings, women often bear disproportionate responsibility for household survival, including managing scarce finances, securing childcare, wage discrimination and limited property rights, trapping them in cycles of dependency and deprivation that fuel anxiety [31–33, 46].

Our findings also indicated that women without formal education showed higher levels of anxiety, which aligns with global evidence [47–49]. Education often equips individuals with critical problem-solving skills, builds self-efficacy, and encourages healthier lifestyle choices that collectively help to mitigate stress and anxiety [50, 51]. A study found that an additional year of schooling reduced the likelihood of reporting any anxiety symptoms by 9.8% [50]. On the other hand, women without any formal education are more likely to be financially reliant, which can lead to restricted control over decision-making and life events, and hence contribute to feelings of insecurity, vulnerability, and heightened anxiety [52, 53]. In line with the finding, this study also indicates that women who discontinued their studies after marriage faced higher levels of anxiety symptoms. This observation is supported by research from India, which revealed that women often face restricted autonomy in career-related decisions, relying heavily on their husbands and extended family for support [54]. Additionally, societal norms and familial expectations frequently act as barriers, preventing many women from continuing their education after marriage [55].

Apart from that, our findings stated that women without a smartphone experienced higher levels of anxiety. Limited access to smartphones may restrict opportunities for communication, information gathering, social interactions, and access to mental health resources, which are crucial for coping with anxiety [56, 57]. In contrast, evidence suggests that smartphone usage can contribute to anxiety, establishing consistent links between excessive smartphone use and increased anxiety levels [58–60]. However, this study also revealed that women who had never used the internet exhibited greater anxiety symptoms. Research indicates significant gender disparities in technological access and use across South Asia, further entrenching social exclusion [61]. In addition, digital exclusion is particularly common in Bangladesh, where women face greater barriers to ICT access and skills development [62]. Contrarily, a prior study conducted on Indian women demonstrated a significant association between internet addiction and heightened levels of psychological distress [63].

Moreover, as the finding suggests, employed women showed an elevated level of anxiousness. A study conducted in Iran highlighted that the acceptance of multiple roles; maternal, marital, and professional, creates conflicting expectations from children, spouses, family, and society [64]. Furthermore, past studies also highlighted that unequal caregiving responsibilities, workplace inequities, and inadequate support may contribute to anxiety among working women [65, 66]. Although employment can provide empowerment, structural barriers often undermine its advantages; thus, further qualitative study is recommended why employed women are more likely to feel anxiety in the context of Bangladesh.

Our study also found that women who had their first sexual encounter before the age of 18 report higher anxiety levels. This observation aligns with existing research that highlights the association between early sexual initiation (ESI) and adverse mental health outcomes [67–69]. This could be due to multiple factors, including societal stigma, feelings of regret, or guilt, and the challenges of navigating complex emotional experiences at a young age [70]. Due to potential negative consequences such as unsafe abortions, sexually transmitted infections (STIs), and unwanted pregnancies, ESI is often regarded as risky sexual behavior [71–73]. Notably, a nationwide cross-sectional study conducted in Bangladesh highlighted that the prevalence of ESI among female adolescents is a pressing public health concern, contributing to long-term mental health challenges [71]. In addition, as majority of the population in Bangladesh are predominantly Muslim, where ESI without marriage is considered a severe sin, so if it occurs without marriage, it could potentially increase the anxiety level in later age even after marriage, although it was impossible in this study to establish whether they were married or unmarried during ESI.

Our research also revealed that women who had their first childbirth before the age of 18 reported higher anxiety levels. This observation aligns with existing research that underscores the vulnerability of teenage mothers, which can be attributed to hormonal fluctuations during adolescence, unplanned parenthood, and societal stigma [74, 75]. Research also highlights those adolescent mothers are more likely to experience economic hardship and reside in socially disadvantaged communities, which negatively impact maternal mental health and parenting outcomes [76, 77].

Evidence from this research suggested that women who never used any contraception to delay or avoid pregnancy reported higher levels of anxiety. This observation aligns with existing research demonstrating lower rates of contraceptive use and reliance on less effective methods that are associated with psychological distress [78]. This is possibly due to concerns about unplanned pregnancies and reduced reproductive autonomy. Interestingly, some studies have linked the use of contraceptives to increase the extent of anxiety and depression [79, 80].

This study also noted that women who reported being pressured by their husbands or family members to become pregnant experienced higher levels of anxiety. This is consistent with studies suggesting that women have limited control over family planning decisions, such as when to have children, the number of children, and contraceptive use [81, 82]. A study conducted in India revealed that one in five women pressurized from their in-laws to conceive immediately after marriage, which can contribute to increased mental distress [83].

Additionally, women whose husbands had no formal education reported higher anxiety score. Evidence from Pakistan revealed that women whose husbands were unemployed exhibited significantly higher levels of depression and anxiety [84]. This may be attributed to the socioeconomic disadvantages associated with lower educational attainment, including limited employment opportunities and reduced household income. Women whose husbands were older than 50 years exhibited higher levels of anxiety. This may be due to concerns about health issues, financial instability, and the additional caregiving responsibilities that often come with an older spouse [85, 86].

Our findings also demonstrated that women who made decisions regarding healthcare visits alone or in collaboration with their partners reported greater levels of anxiety. This could reflect the stress and pressure associated with healthcare decision-making, which may carry significant emotional or financial implications, particularly when women make decisions solely [87]. Similarly, decisions about visiting relatives' houses, whether made alone or in collaboration with their spouse, were linked to higher levels of anxiety in women. This may be due to the added stress of balancing family obligations, social expectations, and potential interpersonal dynamics, which can contribute to feelings of uncertainty and pressure [88, 89].

In addition to the socioeconomic and demographic predictors discussed, it is crucial to interpret anxiety symptoms within the cultural context of Bangladesh. Cultural norms surrounding gender roles, emotional expression, and marital expectations may significantly influence how women perceive and report anxiety symptoms [90]. Research indicates that mental health stigma in rural areas often leads to somatization of distress, where individuals express psychological issues through physical symptoms [91]. Notably, access to mental health care in Bangladesh is predominantly concentrated in urban areas, which may further exacerbate disparities in diagnosis and treatment between urban and rural populations [92]. Furthermore, in the Bangladeshi cultural context, married women are often expected to prioritize familial responsibilities and suppress personal emotional distress. This may lead them to internalize anxiety symptoms, making them less likely to openly acknowledge or express emotional distress [93, 94]. As such, cultural norms and the structure of the healthcare system may influence both the presentation and reporting of anxiety symptoms. These factors could limit the effectiveness of standardized tools like the GAD-7, which may not fully capture culturally nuanced expressions of psychological distress.

### 4.1. Strengths and Limitations

The key strength of this study lies in the generalizability of its findings to Bangladesh, as it utilizes nationally representative data covering a wide geographic area. Additionally, the use of robust statistical methods, including ANOVA and stepwise multiple regression, enhances the study's reliability. However, certain limitations should be acknowledged. The cross-sectional design restricts causal inference, as both outcome and predictor variables were collected simultaneously. Furthermore, some biological and behavioral variables were not clinically diagnosed, requiring readers to interpret the results with caution. Last, the outcome measures were based on self-reports from the 2 weeks preceding the survey, which may introduce recall bias. Moreover, some variables such as smoking, alcohol use, sudden death of husband, father/mother, children or close relatives, poor health conditions which were found related to anxiety among married women, this study could not include them due to unavailability of the variables in the main database. Therefore, we recommend further qualitative study to bring a real picture and the nexus of anxiety among reproductive women because we want to believe that some data really cannot be obtained quantitatively, which requires qualitative investigation, and further incorporating aforesaid variables could also help to establish real casual inference for quantitative research.

### 4.2. Policy Recommendations

Because mental health issues such as anxiety and depression are stigmatized in Bangladeshi society [95, 96], targeted initiatives should be taken at the individual, community, and government levels. Based on the findings of this study, several policies can be implemented to alleviate anxiety among ever-married women in Bangladesh.

First, empower women's agency and independence so they can make reproductive choices themselves and are not pressured by others to do so. It involves creating awareness about the rights of women, speaking out on traditional gender roles causing emotional stress, educating people about it, and peer support to reduce social stigma.

Second, develop targeted strategies for improving the socioeconomic conditions of married women, specifically economic empowerment opportunities via skill development and microfinance, and better education facilities for them and their spouses. Additionally, allocate financial support and access to resources for economically disadvantaged women, prioritizing those living in rural areas and with low income.

Thirdly, provide specialized counseling services aimed at women who are more prone to anxiety. To facilitate this, mental health counseling at the community level should be free/subsidized and accessible to women, particularly those who are at risk and new mothers. In addition, extend marital and family counseling services to couples to minimize marital conflict and family pressure.

Fourthly, improve the healthcare delivery system so that women have better access to medical services for their reproductive health, including the right to choose whether or not they want to have children, the timing of giving birth, and the appropriate method of contraception use, etc. Moreover, the integration of mental health with the general healthcare system, including screening for anxiety and depression, and training healthcare workers to recognize and manage anxiety are essential.

Finally, steps to be taken at the government level to improve the overall mental health of all citizens. In this regard, some active initiatives are already underway, such as passing a new Mental Health Act in 2018 replacing the antiquated Lunacy Act of 1912, a new Mental Health Policy in 2022 emphasizing community-based services and support, and the National Mental Health Strategic Plan 2020–2030.

## 5. Conclusion

This study highlights the significant association between socioeconomic factors and anxiety among ever-married women in Bangladesh. The findings reveal that financial constraints, education level, employment status, and autonomy in decision-making play crucial roles in shaping mental health outcomes. Women with limited economic resources, lower education, and restricted agency over household decisions are more likely to experience heightened anxiety. Additionally, structural gender norms and societal expectations contribute to the psychological burden faced by married women, reinforcing existing mental health disparities. Given these insights, policy interventions should prioritize financial empowerment, access to education, and mental health support tailored to women's needs. Addressing these issues through targeted initiatives can improve overall well-being and promote gender equity in mental health. Future studies should explore the long-term psychological impact of anxiety on married women's overall well-being and examine the effectiveness of gender-sensitive mental health interventions in patriarchal societies like Bangladesh. Moreover, future studies should incorporate multilevel modeling or cluster-adjusted standard errors to account for intra-cluster correlation arising from shared sociocultural and environmental factors.

## Figures and Tables

**Table 1:** Construct of independent variables.

Code	Independent variables	Categories
V1	Age	15–24 years, 25–34 years, 35–49 years
V3	Education	No education, primary, secondary/higher
V4	Household wealth status	Poor, middle, rich
V5	Religion	Islam, Hinduism/other religion
V6	Place of residence	Urban, rural
V7	Respondent currently employed	No, yes
V8	Age of having first sex	<18, ≥18
V9	Age of first birth	<18, ≥18
V10	Own mobile phone	No, yes
V11	Own smartphone	No, yes
V12	Ever used internet	No, yes
V13	Ever had a terminated pregnancy	No, yes
V14	Ever used anything to delay or avoid getting pregnant	No, yes
V15	Decision on contraceptive use	Respondent alone/with partner, husband/partner alone, other interfere
V16	Decision on health care visit	Respondent alone/with partner, husband/partner alone, other interfere
V17	Decision on visiting relative house	Respondent alone/with partner, husband/partner alone, other interfere
V18	Husband education	No education, primary, secondary/higher
V20	Husband or family member pressured respondent to become pregnant	No, yes
V21	Continued studying after marriage	No, yes
V22	Continued work after marriage	No, yes

**Table 2:** General characteristics of the study participants.

Variables	Frequency	Percentage (%)
Age		
15–24 years	1029	3.6
25–34 years	10,073	35.3
35–49 years	17,435	61.1
Education		
No education	3710	13.0
Primary	7375	25.8
Secondary/higher	17,452	61.2
Household wealth status		
Poor	10,767	37.7
Middle	5618	19.7
Rich	12,152	42.6
Religion		
Islam	25,545	89.5
Hinduism/other religion	2992	10.5
Place of residence		
Urban	9973	34.9
Rural	18,564	65.1
Respondent currently employed		
No	13,281	69.9
Yes	5706	30.1
Age of having first sex		
<18	12,417	65.4
≥18	6570	34.6
Age of first birth		
<18	9962	38.9
≥18	15,658	61.1
Own mobile phone		
No	8801	30.8
Yes	19,736	69.2
Own smartphone		
No	10,575	53.6
Yes	9161	46.4
Ever used internet		
No	13,385	70.5
Yes	5602	29.5
Ever had a terminated pregnancy		
No	22,097	77.4
Yes	6440	22.6
Ever used anything to delay or avoid getting pregnant		
No	2906	15.3
Yes	16,081	84.7
Decision on contraceptive use		
Respondent alone/with partner	17,053	89.8
Husband/partner alone	1684	8.9
Other interfere	250	1.3
Decision on health care visit		
Respondent alone/with partner	14,100	74.3
Husband/partner alone	4368	23.0
Other interfere	519	2.7
Decision on visiting relative house		
Respondent alone/with partner	14,000	73.7
Husband/partner alone	3871	20.4
Other interfere	1116	5.9
Husband Education		
No education	4011	21.1
Primary	5306	27.9
Secondary/higher	9670	50.9
Husband or family member pressured respondent to become pregnant		
No	18,400	96.9
Yes	587	3.1
Continued studying after marriage		
No	10,108	80.7
Yes	2410	19.3
Continued work after marriage		
No	808	51.4
Yes	765	48.6
Anxiety status		
Minimal anxiety	14,081	74.2
Mild anxiety	4126	21.7
Moderate anxiety	608	3.2
Severe anxiety	172	0.9

**Table 3:** Level of anxiety among the married women in Bangladesh (*n*, %).

Sl. no.	Questions (items)	Never	Rarely	Often	Always
1	Last 2 weeks: feeling nervous, anxious, on edge	8021 (41.7)	9087 (47.2)	1360 (7.1)	764 (4.0)
2	Last 2 weeks: not able to stop/control worrying	12,645 (65.7)	5652 (29.4)	635 (3.3)	298 (1.5)
3	Last 2 weeks: worrying about things	10,349 (53.8)	7325 (38.1)	1134 (5.9)	423 (2.2)
4	Last 2 weeks: trouble relaxing	13,960 (72.6)	4394 (22.8)	629 (3.3)	250 (1.3)
5	Last 2 weeks: restless and hard to sit still	14,122 (73.4)	4335 (22.5)	565 (2.9)	206 (1.1)
6	Last 2 weeks: become easily annoyed/irritable	10,804 (56.2)	7206 (37.5)	875 (4.5)	345 (1.8)
7	Last 2 weeks: feeling afraid as something awful might happen	16,136 (83.9)	2598 (13.5)	362 (1.9)	136 (0.7)

*Note:* The 2022 BDHS utilized the validated Water Glass Pictorial Scale to assess symptom intensity. This scale uses visual representations, where an empty glass signifies “never,” a one-fourth-full glass represents “rarely,” a half-full glass indicates “often,” and a three-fourths-full glass denotes “always”.

**Table 4:** One-way ANOVA results against total score of GAD.

Variables	Frequency	Mean GAD score	SD	Sig.
Age				
15–24 years	704	2.72 (2.50–2.95)	3.043	0.011*⁣*^*∗*^
25–34 years	6739	3.03 (2.95–3.10)	3.079	
35–49 years	11,544	3.08 (3.02–3.14)	3.155	
Education				
No education	2432	3.72 (3.59–3.85)	3.309	<0.001*⁣*^*∗∗∗*^
Primary	4893	3.37 (3.28–3.46)	3.239	
Secondary/higher	11,662	2.77 (2.72–2.83)	3.000	
Household wealth status				
Poor	7084	3.27 (3.20–3.35)	3.168	<0.001*⁣*^*∗∗∗*^
Middle	3786	3.09 (2.98–3.19)	3.162	
Rich	8117	2.83 (2.77–2.90)	3.054	
Religion				
Islam	17,015	3.10 (3.05–3.15)	3.160	<0.001*⁣*^*∗∗∗*^
Hinduism/other religion	1972	2.61 (2.49–2.73)	2.763	
Place of residence				
Urban	6622	2.99 (2.92–3.06)	3.041	0.059
Rural	12,365	3.08 (3.02–3.13)	3.168	
Respondent currently employed				
No	13,281	3.00 (2.94–3.05)	3.116	<0.001*⁣*^*∗∗∗*^
Yes	5706	3.17 (3.09–3.25)	3.142	
Age of having first sex				
<18	12,417	3.13 (3.08–3.19)	3.147	<0.001*⁣*^*∗∗∗*^
≥18	6570	2.89 (2.81–2.96)	3.075	
Age of first birth				
<18	6723	3.22 (3.14–3.30)	3.195	<0.001*⁣*^*∗∗∗*^
≥18	10,332	3.03 (2.97–3.09)	3.104	
Own mobile phone				
No	5834	3.07 (2.99–3.15)	3.125	0.524
Yes	13,153	3.04 (2.98–3.09)	3.124	
Own smartphone				
No	7011	3.27 (3.20–3.35)	3.177	<0.001*⁣*^*∗∗∗*^
Yes	6142	2.77 (2.70–2.85)	3.042	
Ever used internet				
Never	13,385	3.21 (3.15–3.26)	3.161	<0.001*⁣*^*∗∗∗*^
Yes	5602	2.67 (2.59–2.75)	3.002	
Ever had a terminated pregnancy				
No	14,528	2.91 (2.86–2.96)	3.049	<0.001*⁣*^*∗∗∗*^
Yes	4459	3.48 (3.38–3.58)	3.322	
Ever used anything to delay or avoid getting pregnant				
No	2906	3.05 (2.93–3.17)	3.238	0.947
Yes	16,081	3.05 (3.00–3.10)	3.104	
Decision on contraceptive use				
Respondent alone/with partner	17,053	3.03 (2.99–3.08)	3.142	0.134
Husband/partner alone	1684	3.16 (3.02–3.30)	2.930	
Other interfere	250	3.28 (2.89–3.68)	3.188	
Decision on health care visit				
Respondent alone/with partner	14,100	3.13 (3.08–3.18)	3.183	<0.001*⁣*^*∗∗∗*^
Husband/partner alone	4368	2.89 (2.80–2.98)	2.929	
Other interfere	519	2.19 (1.94–2.44)	2.937	
Decision on visiting relative house				
Respondent alone/with partner	14,000	3.14 (3.09–3.19)	3.191	<0.001*⁣*^*∗∗∗*^
Husband/partner alone	3871	2.98 (2.89–3.08)	2.996	
Other interfere	1116	2.11 (1.97–2.26)	2.499	
Husband education				
No education	4011	3.59 (3.49–3.69)	3.311	<0.001*⁣*^*∗∗∗*^
Primary	5306	3.15 (3.07–3.24)	3.170	
Secondary/higher	9670	2.76 (2.71–2.82)	2.983	
Husband or family member pressured respondent to become pregnant				
No	18,400	3.00 (2.96–3.05)	3.075	<0.001*⁣*^*∗∗∗*^
Yes	587	4.47 (4.13–4.81)	4.155	
Continued studying after marriage				
No	10,108	3.06 (3.00–3.13)	3.196	<0.001*⁣*^*∗∗∗*^
Yes	2410	2.53 (2.42–2.64)	2.761	
Continued work after marriage				
No	808	3.56 (3.32–3.81)	3.533	0.069
Yes	765	3.29 (3.06–3.52)	3.230	

Abbreviation: SD, standard deviation.

*⁣* ^*∗∗∗*^Significant at <0.001%.

*⁣* ^*∗∗*^Significant at 0.001%.

*⁣* ^*∗*^Significant at ≤0.05%.

**Table 5:** Stepwise multiple regression.

Predictors (code)	*R* square	*R* square change	*F* change	Sig.
V13	0.068	0.068	13.213	<0.001
V13, V20	0.106	0.038	12.112	<0.001
V13, V20, V3	0.134	0.028	12.781	<0.001
V13, V20, V3, V5	0.153	0.019	7.097	0.018
V13, V20, V3, V5, V21	0.165	0.012	4.325	0.030
V13, V20, V3, V5, V21, V19	0.173	0.007	4.129	0.033

*Note:* Anxiety status is the dependent variable. V13 = Ever had a terminated pregnancy; V20 = Husband or family member pressured respondent to become pregnant, V3 = Respondent's education, V5 = Religion, V21 = Continued study after marriage, V19 = Husband education. Sig. = Level of significance.

## Data Availability

The data that support the findings of this study are available in the Demographic and Health Surveys Program at https://dhsprogram.com. These data were derived from the following resources available in the public domain: Bangladesh: Standard DHS, 2022, https://dhsprogram.com/methodology/survey/survey-display-584.cfm.
